# Adiponectin improves long-term potentiation in the 5XFAD mouse brain

**DOI:** 10.1038/s41598-019-45509-0

**Published:** 2019-06-20

**Authors:** Ming Wang, Jihoon Jo, Juhyun Song

**Affiliations:** 1 Department of Biomedical Sciences, BK21 PLUS Center for Creative Biomedical Scientists at Chonnam National University, Research Institute of Medical Sciences, Chonnam National University Medical School, Gwangju, 501-757 Republic of Korea; 2 NeuroMedical Convergence Lab, Biomedical Research Institute, Chonnam National University Hospital, Jebong-ro, Gwangju 501-757 Republic of Korea; 3 Department of Neurology, Chonnam National University Medical School, Gwangju, 501-757 Republic of Korea; 4 Department of Anatomy, Chonnam National University Medical School, Hwasun, 58128 Jeollanam-do Republic of Korea

**Keywords:** Long-term memory, Neurodegeneration, Alzheimer's disease

## Abstract

Adiponectin is an adipokine that regulates apoptosis, glucose and lipid metabolism, and insulin sensitivity in metabolic diseases. As recent studies have associated changes in adipokines and other metabolites in the central nervous system with a risk for Alzheimer’s disease (AD), we investigated the effects of adiponectin treatment on hippocampal cells in the 5XFAD mouse model of AD and neuronal SH-SY5Y cells under amyloid beta toxicity. Adiponectin treatment reduced levels of cleaved caspase 3 and nuclear factor kappa-light-chain-enhancer of activated B cells (NF-κB) apoptosis signalling and decreased glycogen synthase kinase 3 beta (GSK3β) activation. Moreover, adiponectin treatment triggered long-term potentiation in the hippocampi of 5XFAD mice, which was associated with reduced expression of *N*-methyl-d-aspartate and its receptor as well as surface expression of the α-amino-3-hydroxy-5-methylisoxazole-4-propionic acid receptor. These findings suggest that adiponectin inhibits neuronal apoptosis and inflammatory mechanisms and promotes hippocampal long-term potentiation. Thus, adiponectin exhibits beneficial effect on hippocampal synaptic plasticity in Alzheimer’s disease mouse model.

## Introduction

Adiponectin is the most abundant adipokine secreted by adipose tissue^1^; it circulates throughout the body and acts as an endocrine hormone to regulate glucose and lipid metabolism^2^, insulin resistance, and inflammation^3^. A recent study found that type 2 diabetes patients with cognitive decline have low serum levels of adiponectin^4^. Other recent studies have associated low levels of adiponectin with reduced grey matter and hippocampal volumes and impaired glucose metabolism, which are related to Alzheimer’s disease (AD) pathophysiologies^5,6^. Reduced serum levels of adiponectin are also associated with mild cognitive impairment in AD patients^7^ and are exhibited by a transgenic amyloid precursor protein mouse model of AD^8^.

Adiponectin can exert effects in brain regions that highly express its receptors, AdipoR1 and AdipoR2, including the hypothalamus, cerebral cortex and hippocampus^9^, a region associated with learning and memory^10^. Cognitive decline in AD is attributed to synaptic dysfunction in the hippocampus^11^, related to a loss of long-term potentiation (LTP), a molecular mechanism of hippocampal synaptic plasticity^12–14^. Adiponectin treatment was shown to enhance synaptic function^15^ and promote presynaptic release^16^, and increases in circulating adiponectin levels alleviate memory dysfunction in models of dementia^5,17^. To evaluate the effects of adiponectin on hippocampal LTP as well as the mobility of cell surface ionotropic glutamate receptors in AD, we utilised a mouse model harbouring five familial AD mutations, the 5XFAD mouse. 5XFAD mice co-overexpress human amyloid precursor protein and presenilin-1, resulting in an additive overproduction of amyloid beta (Aβ) and early stages of AD-related neuropathology and memory deficits^18^. Excess Aβ peptide has been shown to impair hippocampal LTP, leading to memory deficits and cognitive decline in AD^19^. Our results indicate that adiponectin has therapeutic potential to improve hippocampal function in AD.

## Results

### Adiponectin enhances hippocampal plasticity in 5XFAD mice

**Figure 1:**
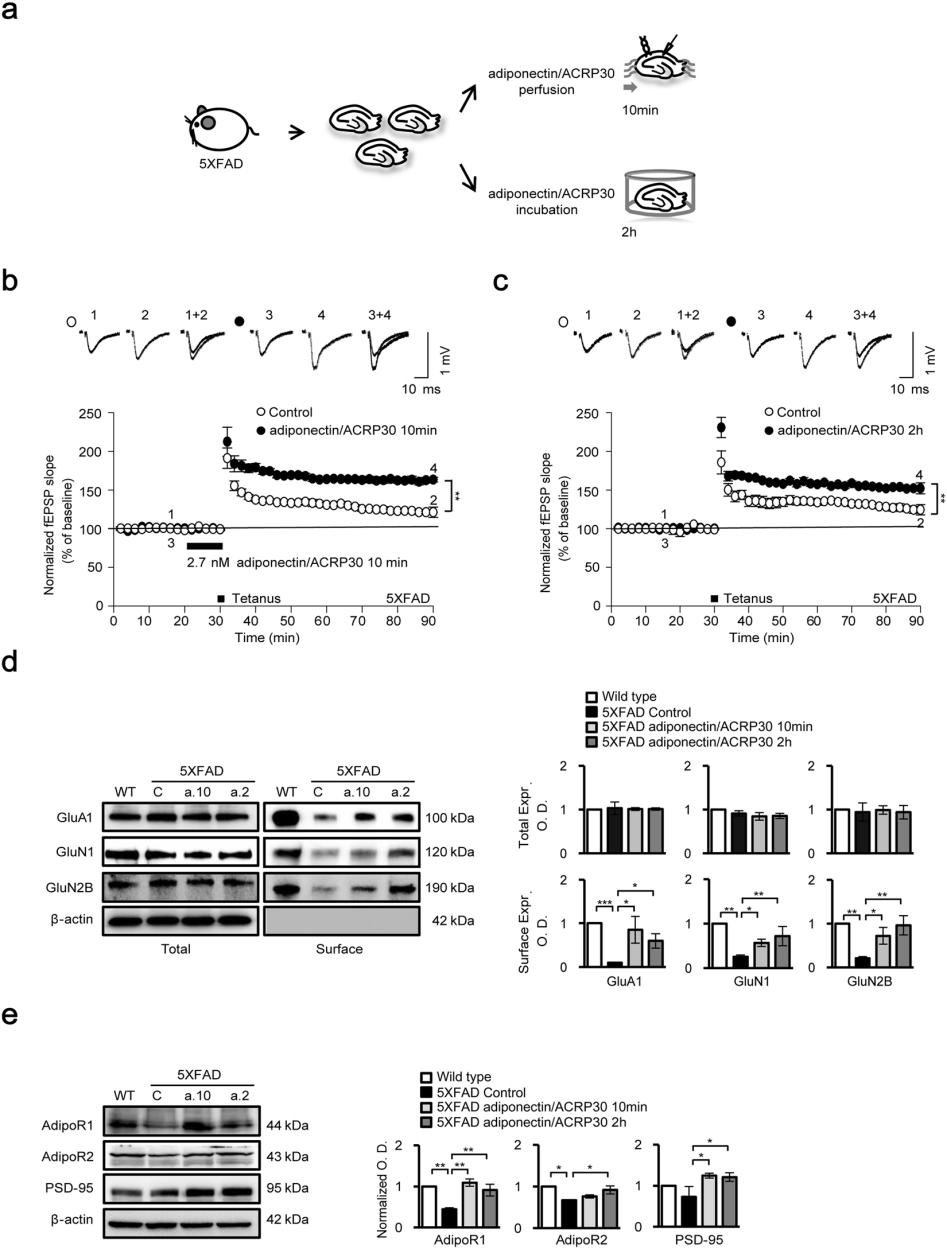
Electrophysiological recordings in the CA1 hippocampal region, and ionotropic glutamate receptor, AdipoR1, AdipoR2 and PSD-95 protein expression after adiponectin/ACRP30 treatment. (**a**) Schematic diagrams of the experimental procedure and field recordings in hippocampal slices. (**b**,**c**) High-frequency stimulation (HFS; two trains of 100 Hz, 100 pulses) failed to induce LTP in 5XFAD mice (*n* = 4). However, HFS induced robust LTP in slices following adiponectin/ACRP30 perfusion (10 min) and incubation (2 h) (closed circles). (**d**) *In vitro* surface expression of GluA1, GluN1 and GluN2B decreased in the 5XFAD mouse hippocampus compared with that in wild-type mice; however, these decreases were reversed by adiponectin/ACRP30 treatment under both conditions (*n* = 3). No change in GluA1 (AMPA receptor subunit), GluN1 (NMDA receptor subunit) or GluN2B (NMDA receptor subunit) expression was observed (*n* = 3). (**e**) AdipoR1, AdipoR2 and PSD-95 protein levels were decreased in 5XFAD mouse hippocampus compared with those in wild-type mice; however, adiponectin/ACRP30 significantly increased protein levels under both treatment conditions. Data are expressed as means ± SEMs. *p < 0.05, **p < 0.001, ***p < 0.0001; adiponectin/ACRP30 perfusion, 2.7 nM adiponectin/ACRP30 perfusion for 10 min; adiponectin/ACRP30 incubation, 2.7 nM adiponectin/ACRP30 incubation for 2 h; a.10, adiponectin/ACRP30 treatment for 10 min; a.2, adiponectin/ACRP30 treatment for 2 h.

To assess the effects of adiponectin on hippocampal plasticity, we performed field recordings using hippocampal slices from 5- to 6-month-old 5XFAD mice. Slices were treated with 2.7 nM adipocyte complement-related protein of 30 kDa (adiponectin/ACRP30), which approximates the physiological concentration of adiponectin in human cerebrospinal fluid (CSF)^20^. Hippocampal slices were perfused with artificial CSF (aCSF) containing adiponectin/ACRP30 either for 10 min prior to a tetanus (two trains of 100 Hz, 100 pulses) before returning to normal aCSF, as in a recent study^16^, or for 2 h prior to the field recordings to examine the long-term effects of adiponectin/ACRP30 on LTP induction and molecular modulation (Fig. 1a). LTP induction was assessed by recording field excitatory postsynaptic potentials (fEPSPs). Whereas high-frequency stimulation failed to produce LTP in the CA1 region of 5XFAD hippocampus (control, 121% ± 2%; control, 124% ± 7%; *n* = 4), treatment with adiponectin/ACRP30 rescued the LTP impairment (10 min perfusion, 163% ± 3% of baseline; 2 h incubation, 152% ± 7%; *n* = 6) (Fig. 1b,c).

To investigate whether this rescue involved alterations in the surface expression of glutamate receptors, we performed biotinylation pull-down assays for α-amino-3-hydroxy-5-methylisoxazole-4-propionic acid (AMPA) receptors and *N*-methyl-d-aspartate (NMDA) receptors. Our results indicated that adiponectin/ACRP30 treatment (perfusion or incubation) promoted the surface expression of the NMDA receptor subunits GluN1 and GluN2B and the AMPA receptor subunit GluA1 in the 5XFAD mouse hippocampus compared with levels in the controls (Fig. 1d). However, the total expression of NMDA and AMPA receptors did not change. Western blot analyses revealed that AdipoR1 and AdipoR2 expression was lower in hippocampal tissues from 5XFAD mice than in controls (Fig. 1e). Furthermore, we observed a dramatic change in AdipoR1 versus AdipoR2 expression (Fig. 1e). Therefore, we suggest that the effects of adiponectin/ACRP30 in the AD brain may be mainly through AdipoR1. In line with the observed rescue of LTP, treatments with adiponectin/ACRP30 also increased the expression of postsynaptic density protein 95 (PSD-95) in 5XFAD mouse hippocampus compared with that in controls (Fig. 1e).

### Adiponectin alters GSK3β signalling and cytokine levels in the hippocampus

**Figure 2:**
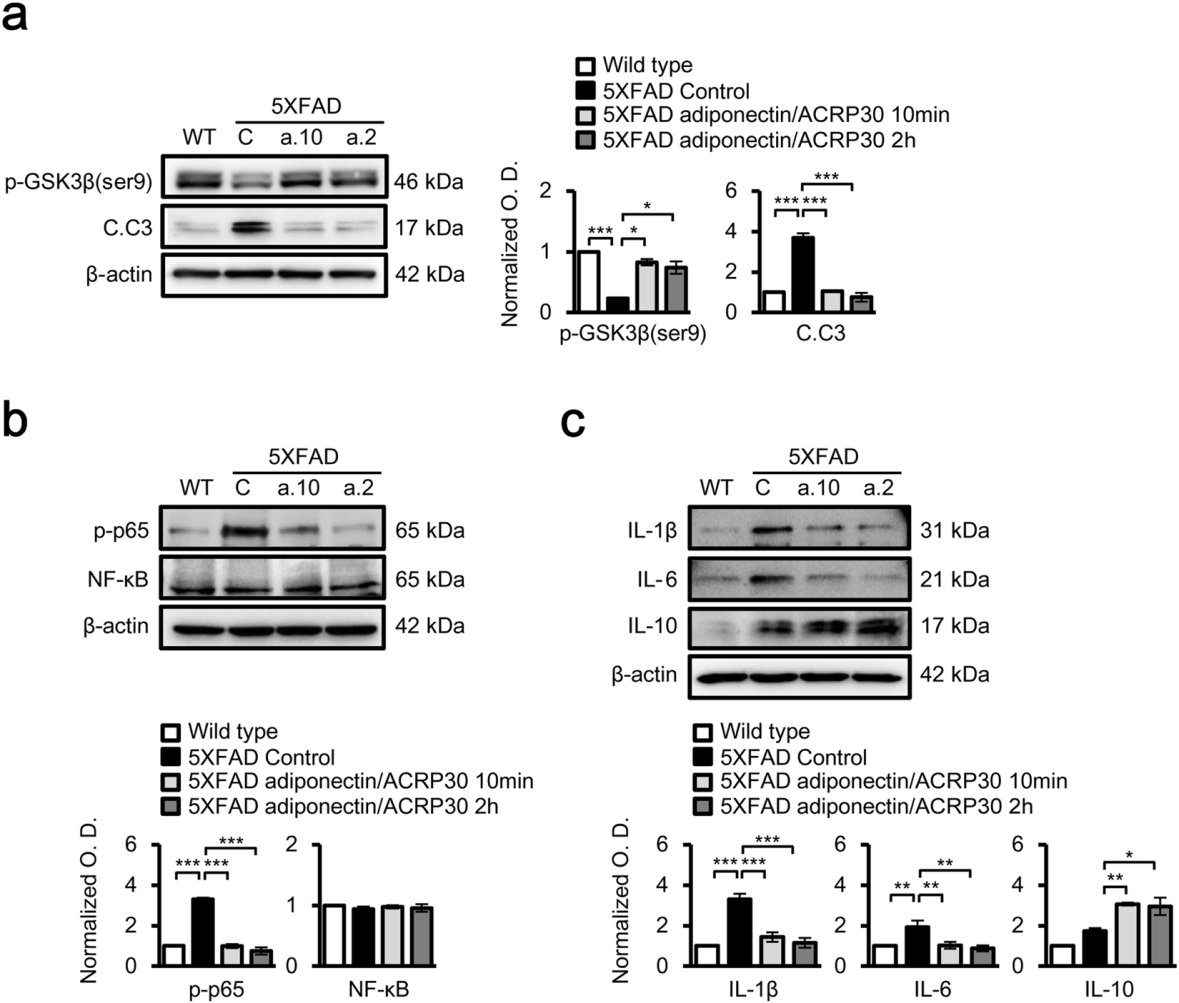
Adiponectin/ACRP30 alleviates aberrant GSK3β and NF-κB signalling in the 5XFAD mouse hippocampus. (**a**) GSK3β activation and cleaved caspase 3 protein levels were increased in 5XFAD mouse hippocampus compared with those in wild-type mice; these were alleviated by adiponectin/ACRP30 treatment for 10 min and 2 h. (**b**) Phosphorylated p65 (p-p65) protein levels were significantly increased in 5XFAD mouse hippocampus compared with those in the control; this increase was suppressed by adiponectin/ACRP30 treatment for 10 min and 2 h. (**c**) Analysis of IL-1β, IL-6 and IL-10 expression by Western blotting (*n* = 4 in each group). Data are expressed as means ± SEMs. *p < 0.05, **p < 0.001, ***p < 0.0001; a.10 and a.2 indicate 2.7 nM adiponectin/ACRP30 treatment for 10 min and 2 h, respectively; C, 5XFAD control mouse hippocampus.

Western blotting showed increased phosphorylation (Ser9) of glycogen synthase kinase 3 beta (GSK3β) and reduced levels of cleaved caspase 3 (Fig. 2a) and phosphorylated tau (p-tau; Ser404) (Supplementary Fig. S1a) in the hippocampal tissues of 5XFAD mice compared with those in controls following adiponectin/ACRP30 treatment. However, adiponectin/ACRP30 treatment did not alter the expression of Aβ_42_ in 5XFAD mouse brain (Supplementary Fig. S1b). 5XFAD mouse brain also showed higher phosphorylation of the p65 subunit of nuclear factor kappa-light-chain-enhancer of activated B cells (NF-κB) than controls, and this increase was ameliorated by adiponectin/ACRP30 treatment (Fig. 2b). Western blotting also revealed that adiponectin/ACRP30 treatment altered the expression of cytokines, such as interleukin-6 (IL-6), IL-1β and IL-10, in the 5XFAD mouse hippocampus. Specifically, adiponectin treatment reduced proinflammatory cytokine levels and enhanced anti-inflammatory cytokine levels in 5XFAD mouse hippocampus (Fig. 2c). These alterations following adiponectin/ACRP30 treatment of hippocampal slices from 5XFAD mouse brain were confirmed by enzyme-linked immunosorbent assays (ELISAs) (Supplementary Fig. S2).

**Figure 3:**
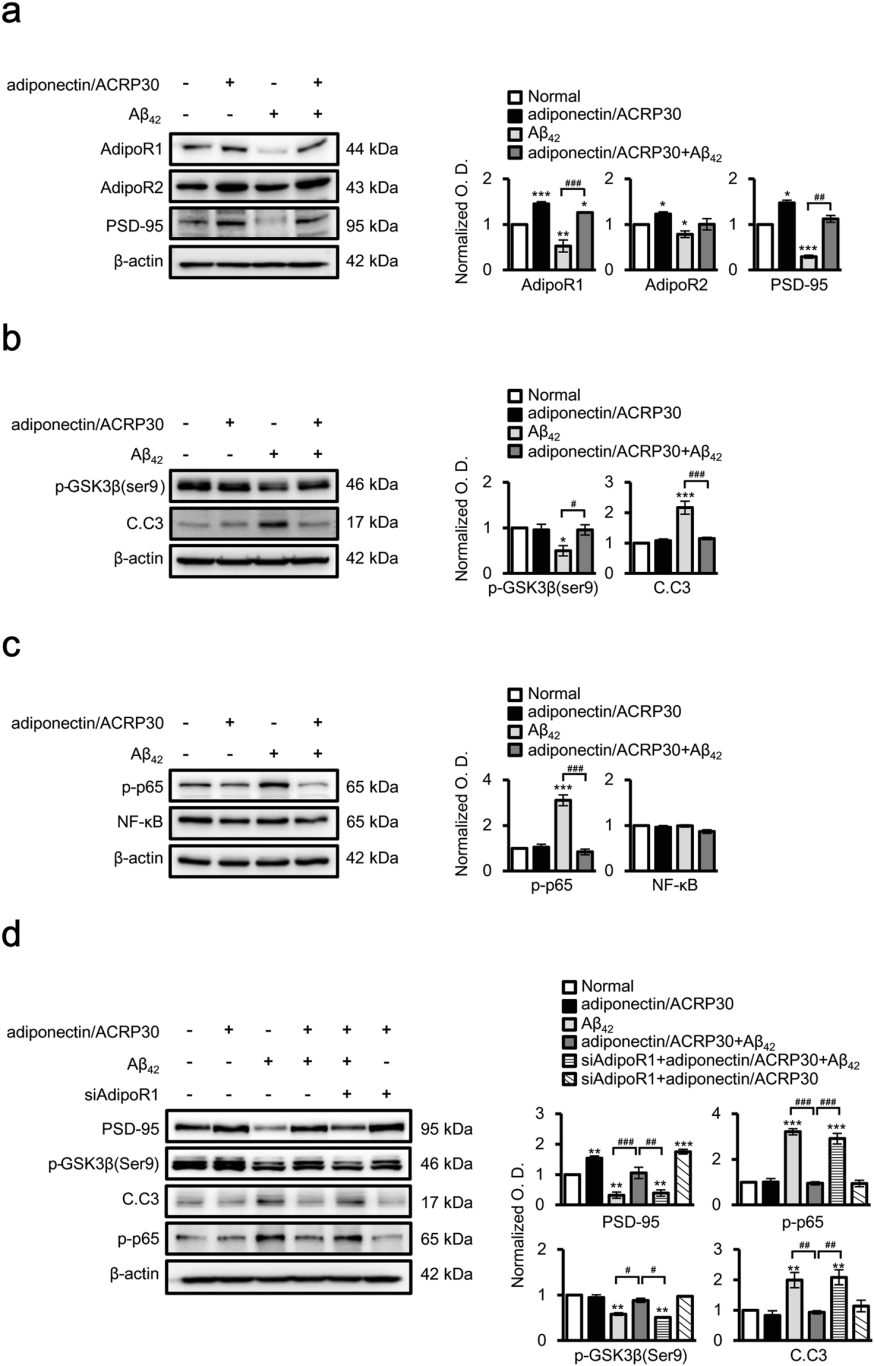
Altered activation of NF-κB and GSK3β signalling in neuronal SH-SY5Y cells under Aβ toxicity by adiponectin/ACRP30 treatment. Western blot analysis of protein expression levels in neuronal SH-SY5Y cells under Aβ_42_ toxicity (**a**–**c**) and following the transfection with AdipoR1-specific siRNAs (**d**). (**a**) AdipoR1, AdipoR2 and PSD-95 protein levels decreased after Aβ_42_ treatment compared with those in the control. However, except for AdipoR2, these decreases were rescued by adiponectin/ACRP30 treatment. (**b**) GSK3β activation and cleaved caspase 3 protein levels increased after Aβ_42_ treatment compared with those in the control but were reversed by adiponectin/ACRP30 treatment. (**c**) p-p65 levels were significantly increased after Aβ_42_ treatment compared with those in the control and were suppressed by adiponectin/ACRP30 treatment. (**d**) Adiponectin treatment prevented the effect of Aβ_42_ toxicity on PSD-95, p-GSK3β (ser9), p-p65 and cleaved caspase 3 protein levels. However, AdipoR1 siRNAs fully reversed these effects. Data are expressed as means ± SEMs. *p < 0.05 compare with control, **p < 0.001 compare with control, ***p < 0.0001 compare with control; ^**#**^*p* < 0.05, ^**##**^*p* < 0.001, ^**###**^*p* < 0.0001; adiponectin/ACRP30, 20 nM treatment for 12 h; Aβ_42_, treatment with 10 μM Aβ_42_ peptide for 24 h in neuronal SH-SY5Y cells; adiponectin/ACRP30 + Aβ_42_, SH-SY5Y cells were treated with adiponectin/ACRP30 (20 nM) for 12 h and then incubated with Aβ_42_ peptide (10 μM) for 24 h; siAdipoR1 + adiponectin/ACRP30 + Aβ_42_, SH-SY5Y cells were transfected with siAdipoR1 (5 μM) for 48 h and then treated with Aβ_42_ and adiponectin/ACRP30; siAdipoR1 + adiponectin/ACRP30, SH-SY5Y cells were transfected with siAdipoR1 (5 μM) for 48 h and then treated with adiponectin/ACRP30; C.C3, cleaved caspase 3.

Finally, to further confirm the effect of adiponectin/ACRP30 treatment on GSK3β and NF-κB signalling, we measured the phosphorylation of GSK3β (ser9) and p65, as well as PSD-95 and cleaved caspase 3 levels, in adiponectin/ACRP30-treated neuron-like cells *in vitro* (Fig. 3). SH-SY5Y cells under Aβ toxicity that were treated with adiponectin/ACRP30 exhibited increased p-GSK3β and PSD-95 expression, but decreased p-p65 expression compared with that in untreated cells (Fig. 3a–c). Furthermore, whereas Aβ toxicity reduced AdipoR1 expression levels, treatment with adiponectin/ACRP30 mitigated this decrease but did not affect the expression of AdipoR2 (Fig. 3a). Cotreatment with adiponectin/ACRP30 also blocked the increase in beta-secretase 1 protein levels in SH-SY5Y cells under Aβ toxicity (Supplementary Fig. S3). To determine if the activation of AdipoR1 contributed to the phosphorylation of GSK3β, AdipoR1 expression was knocked down in SH-SY5Y cells. Cells transfected with AdipoR1-specific siRNAs with adiponectin/ACRP30 had reduced levels of phosphorylated GSK3β and PSD-95, and also increased expression of p-p65and cleaved caspase 3 under conditions of Aβ toxicity (Fig. 3d).

Taken together, the findings presented here reveal that adiponectin/ACRP30 attenuates GSK3β and NF-κB activation in the AD hippocampus, which likely contributes to the suppression of neuroinflammation and restoration of LTP.

## Discussion

The results of the present study highlight three potential therapeutic functions of adiponectin/ACRP30 for treating AD. First, adiponectin/ACRP30 treatment markedly increased the surface expression of NMDA and AMPA receptors in the 5XFAD mouse hippocampus. NMDA receptor activation is decreased in the AD brain compared with that in the normal brain^21^. Moreover, NMDA receptor subunits such as GluN2A and GluN2B mediate LTP induction^22^. Given the critical roles of NMDA receptor activation and AMPA receptor trafficking in LTP induction and expression^23^, our findings suggest that the rescue of LTP in the 5XFAD mouse hippocampus by adiponectin/ACRP30 was mediated by the recruitment of NMDA and AMPA receptors to the cell membrane.

Second, adiponectin/ACRP30 inhibited GSK3β activation, which is associated with impaired hippocampal LTP^24^. GSK3β is highly expressed in the hippocampus and is associated with tau hyperphosphorylation and presenilin-1 phosphorylation in AD^25^. Inhibition of GSK3β activation triggers NMDA receptor activation and LTP induction^26^. Our findings are consistent with those of another study in which adiponectin/ACRP30 modulated GSK3β/β-catenin signalling transduction^27^. Our findings suggest that adiponectin/ACRP30 suppressed the activation of GSK3β and rescued LTP impairment in the 5XFAD hippocampus, resulting in improvement of synaptic deficits.

Third, adiponectin/ACRP30 suppressed neuroinflammation by regulating NF-κB activation in the AD hippocampus. NF-κB is a transcription factor that regulates neuroinflammation in the AD brain and critically controls the progression of AD^28^. For example, the suppression of neuroinflammation was shown to lead to improvements in cognitive function in AD^29^. Our findings are also consistent with those of another study that showed that adiponectin regulates NF-κB activation and inhibits the expression of proinflammatory cytokines^30^.

Collectively, the results from the present study indicate that adiponectin/ACRP30 may potentially be useful to overcome the synaptic impairments in AD brain. Further studies are needed to explore the clinical application of adiponectin/ACRP30 as a promising therapeutic option for AD.

## Methods

### Animal experiments

Male 5-month-old 5XFAD transgenic mice (strain B6SJL-Tg[APPSwFlLon,PSEN1*M146L*L286V]6799Vas/J) were obtained from The Jackson Laboratory (Bar Harbor, ME, USA). Wild-type male C57BL/6 mice (250–300 g, 4 months) were obtained from Koatech (Pyeongtaek, South Korea). Aβ_42_ production was checked in whole brain tissue from animals at 2 months of age. The experiment was carried out in accordance with the recommendations of ’96 Guidance for Animal Experiments’, established by the ‘Animal Ethics Committee’at Chonnam National University, and the protocol was approved by the ‘Animal Ethics Committee’ at Chonnam National University.

### Cell culture and drug treatment

SH-SY5Y neuroblastoma cells (1 × 10^5^ cells/ml) were subcultured in Dulbecco’s modified Eagle’s medium supplemented with 10% foetal bovine serum (Gibco, Grand Island, NY, USA) and 100 μg/ml penicillin-streptomycin (Gibco) for 2 days in a humidified atmosphere of 5% CO_2_ at 37 °C. The medium was then changed to medium supplemented with 1% foetal bovine serum and 5 μM retinoic acid to promote differentiation into neuron-like cells. The cells were then treated with 20 nM adiponectin/ACRP30 (the globular form of adiponectin) (Sigma-Aldrich, St. Louis, MO, USA) for 12 h and/or with 10 μM Aβ_42_ for 24 h. AdipoR1 expression was silenced by incubating the cells for 48 h with 5 μM siRNA (sc-60123; Santa Cruz Biotechnology, Dallas, TX, USA) and Lipofectamine 2000 (Invitrogen, Carlsbad, CA, USA) in Opti-MEM medium for 15 min at room temperature.

### Aβ_42_ preparation

Aβ_42_ oligomers were prepared as previously reported^31^. Synthetic Aβ_42_ oligomers (American Peptide, Sunnyvale, CA, USA) were mixed in 1 mM hexafluoroisopropanol (Sigma-Aldrich) and dried under a gentle stream of nitrogen gas. The peptides were then resuspended in dimethyl sulfoxide at room temperature for 15 min with vortexing. The final stock solution was aliquoted as small volumes and stored at −80 °C. For working solutions, 500–1,000 μl phosphate-buffered saline (Invitrogen) was added to the peptide stock solution for 2 h at room temperature to promote aggregation of the peptide.

### Western blotting

SH-SY5Y cells were washed twice with phosphate-buffered saline and lysed with RIPA buffer (Sigma-Aldrich). Hippocampal tissues slices were incubated for 1 h in oxygenated aCSF with/without adiponectin/ACRP30 treatment and then lysed in ice-cold RIPA buffer (AKR-190; Cell Biolabs, San Diego, CA, USA) with a protease inhibitor cocktail (210205; Cell Biolabs) and homogenised with a pestle on ice. The cell and tissue extracts were centrifuged at 13,000 rpm for 15 min at 4 °C to generate whole-cell extracts, and the protein content in the supernatants was measured with a bicinchoninic acid assay kit (Thermo Scientific, Waltham, MA, USA). Protein samples (30 μg) were separated on 10% SDS-polyacrylamide gels and transferred to polyvinylidene difluoride membranes. After blocking with skim milk in Tris-buffered saline with Tween 20 (20 nM Tris [pH 7.2], 150 mM NaCl and 0.1% Tween 20) for 1 h 30 min at room temperature, the blots were incubated for 16 h at 4 °C with primary antibodies (at 1:1,000 dilutions) specific for cleaved caspase 3, total NF-κB, p-NF-κB p65, p-tau (ser404), p-GSK3β (ser9), PSD-95, IL-1β, IL-6 or IL-10 (Cell Signaling, Danvers, MA, USA), AdipoR1 or AdipoR2 (Santa Cruz Biotechnology) or β-actin (Abcam, Cambridge, MA, USA). The immunoblots were then incubated with specific secondary antibodies (Abcam) for 2 h at room temperature, and the bands were detected via enhanced chemiluminescence (Millipore, Billerica, MA, USA).

### ELISAs

Tumour necrosis factor alpha, IL-6 and IL-1β levels were estimated in lysates from wild-type and 5XFAD mouse hippocampi and in lysates from slices treated for 10 min or 2 h with adiponectin/ACRP30 by ELISAs (R&D Systems) according to the manufacturer’s recommendations. Standard curves were plotted with increasing concentrations of cytokines. Supernatants were incubated in a 96-well microliter plate, and absorbances at 540 nm were measured with a microplate reader.

### Electrophysiology experiments

5XFAD mice were sacrificed by cervical dislocation and then decapitated. The brains were quickly moved to ice-cold aCSF containing (in mM) 124 NaCl, 3 KCl, 26 NaHCO_3_, 1.25 NaH_2_PO_4_, 2 CaCl_2_, 1 MgSO_4_ and 10 glucose. Transverse hippocampal sections (400 μm thick) were obtained using a McIlwain tissue chopper (Mickle Laboratory Engineering Co. Ltd.) and stabilised for 1 h via perfusion with aCSF gassed with a 95% O_2_/5% CO_2_ mixture at room temperature. The slices were then moved to the recording chamber perfused with oxygenated aCSF (28–29 °C) at 2 ml/min. To record extracellular fEPSPs, two stimulating bipolar electrodes (66 μm twisted nichrome wire) were placed on the Schaffer collateral pathway (for LTP input) and the subiculum region (for control input). fEPSPs in the CA1 region were assessed with glass microelectrodes prepared on a micropipette puller (P-1000; Sutter Instrument, Novato, CA, USA) and filled with 3 M NaCl (3–5 MΩ). Stable baseline recordings of responses to low-frequency stimulation (single pulses delivered at 0.016 Hz with a stimulation intensity set to 50–60% of the maximum spike-free fEPSP) were collected for 20–30 min prior to perfusion of adiponectin/ACRP30 or LTP induction. LTP was evoked by two trains of tetanus stimuli (100 Hz for 1 s with a 30 s intertetanus interval). The stimulus intensity during tetanus stimuli was identical to that of the test pulse. After the establishment of the stable baseline for 30 min, fEPSPs were measured for at least 60 min. The slope of the evoked field potential responses was assessed and expressed relative to the normalised preconditioning baseline.

### Immunohistochemistry

Mouse brain sections (10 μm) were mounted on collagen-coated glass slides (Thermo Scientific), fixed in acetone solution for 10 min, washed in Tris-buffered saline and then exposed to methanol for 5 min. Nonspecific labelling was prevented by incubating the sections in 5% bovine serum albumin (Sigma-Aldrich) for 1 h 30 min before incubating for 16 h at 4 °C with the following specific primary antibodies (1:500 dilutions): Aβ_42_ (BioLegend, San Diego, CA, USA). The sections were washed 3 times (5 min each) with phosphate-buffered saline with 0.1% Tween 20 and then incubated for 1 h in the dark with specific fluorescent secondary antibodies (1:500; Invitrogen). All brain sections were counterstained with 1 μg/ml 4′,6-diamidino-2-phenylindole (DAPI; Sigma-Aldrich) and visualised with a confocal microscope (Carl Zeiss, Oberkochen, Germany).

### Biotinylation and streptavidin pull-down assays

Biotinylation and streptavidin pull-down assays were performed to assess the surface expression of AMPA and NMDA receptors in the hippocampus. Hippocampal slices were washed twice in aCSF and incubated in aCSF containing 1 mg/ml sulfo-NHS-SS-biotin for 45 min at 4 °C to label surface membrane proteins. The tissue was then homogenised in lysis buffer containing 25 mM Tris (pH 7.6), 150 mM NaCl, 1% NP-40, 1% sodium deoxycholate, 0.1% SDS, 1 mM NaF and a cocktail of protease inhibitors (Sigma-Aldrich). The lysate was centrifuged at 11,000 × g for 15 min at 4 °C to remove nuclei and cellular debris. The total protein concentration was determined with a bicinchoninic acid assay (Thermo Scientific). A small amount of the lysate was discarded for later whole-cell analysis. Subsequently, 100 μl of streptavidin beads (Thermo Scientific) was added to 500 µg of protein lysate and placed on a rotator at 4 °C for 2 h. The samples were then washed four times in wash buffer (25 mM Tris [pH 7.6], 150 nM NaCl, 0.5% Triton X-100), and all beads were pulled down by centrifugation. Bound proteins were eluted in 2 × SDS reducing buffer and heated at 60 °C for 30 min. The supernatants were transferred to new tubes and heated at 90 °C for 5 min before gel loading.

### Statistical analyses

Statistical analyses were performed using SPSS 21.0 (IBM Corp., Armonk, NY, USA). The data are expressed as the means ± the standard errors of the means (SEMs). Data were analysed using one-way analyses of variance followed by Bonferroni’s *post hoc* multiple comparisons tests. Significant differences were considered at *p* values of < 0.05.

## Supplementary information

supplementary figure legends
