# Supplementary material for: Adiponectin improves long-term potentiation in the 5XFAD mouse brain
Source: Sci Rep. 2019 Jun 20;9:8918. doi: 10.1038/s41598-019-45509-0 (PMC6586823; doi:10.1038/s41598-019-45509-0)
Supplement: Supplementary file 1 — supplementary figure legends [file 41598_2019_45509_MOESM1_ESM.docx]

**Supplementary information**

**Adiponectin improves long-term potentiation in the 5XFAD mouse brain**

Ming Wang, Jihoon Jo*, Juhyun Song*

**
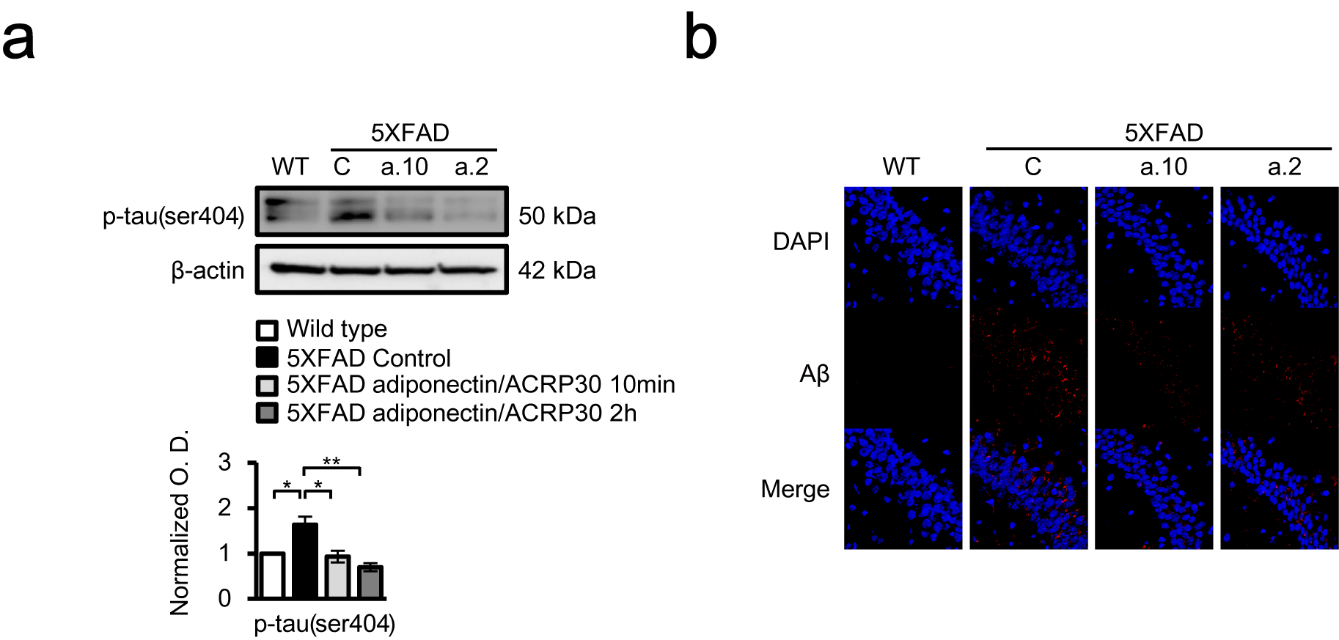
**

**Supplementary Figure 1.** (a) Treatment with adiponectin/ACRP30 for 10 min and 2 h decreased the aberrant p-tau expression in 5XFAD mouse hippocampus. (b) Immunostaining shows that the expression of Aβ_42_ was not considerably changed in 5XFAD mouse brain tissue after adiponectin/ACRP30 treatment. Data are expressed as means ± SEMs. **p* < 0.05, ***p* < 0.001; a.10 and a.2 indicate 2.7 nM adiponectin/ACRP30 treatment for 10 min and 2h, respectively; C, 5XFAD control mouse hippocampus; Aβ, red; DAPI, blue.


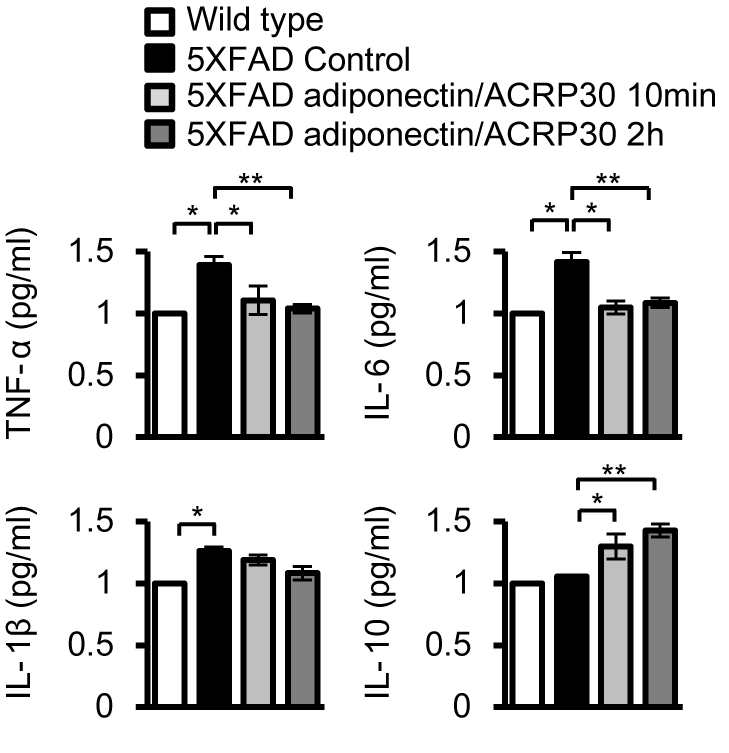


**Supplementary Figure 2.** ELISAs estimating secreted tumour necrosis factor alpha (TNF-α), IL-6 and IL-1β levels in 5XFAD mouse hippocampal slices after adiponectin/ACRP30 treatment. Increased TNF-α and IL-6 levels were significantly suppressed following adiponectin/ACRP30 treatment, but IL-1β levels only changed slightly. Data are expressed as means ± SEMs. **p* < 0.05, ***p* < 0.001.


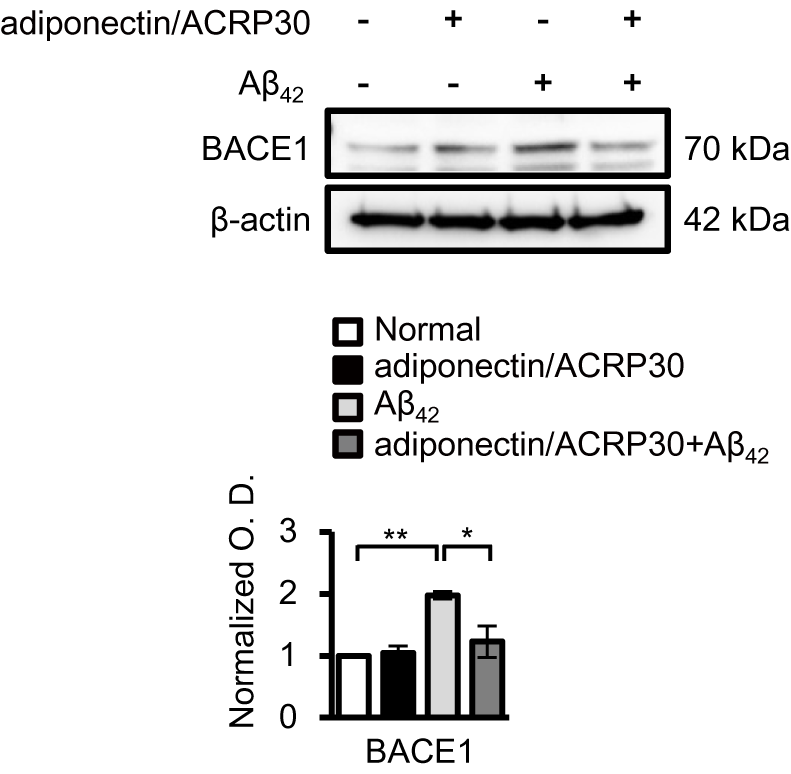


**Supplementary Figure 3.** Beta-secretase 1 (BACE1) protein levels were significantly upregulated by Aβ_42_ treatment compared with those in the control and were suppressed by adiponectin/ACRP30 pretreatment. Data are expressed as means ± SEMs. ***p* < 0.001, ****p* < 0.0001; adiponectin/ACRP30, 20 nM treatment for 12 h; Aβ_42_, treatment with 10 μM Aβ_42_ peptide for 24 h in neuronal SH-SY5Y cells; adiponectin/Acrp30+Aβ_42_, SH-SY5Y cells were treated with adiponectin/ACRP30 (20 nM) for 12 h and then incubated with Aβ_42_ peptide (10 μM) for 24 h.
